# Associations Between Paternal Sensitivity and Cognitive and Behavioural Outcomes in Preterm Children: An Individual Participant Data Meta‐Analysis

**DOI:** 10.1111/cch.70338

**Published:** 2026-09-03

**Authors:** Noa Gueron‐Sela, Karli Treyvaud, Naama Atzaba‐Poria, Jeanie Cheong, Alicia Spittle, Tal Yatziv, Peter J. Anderson, Julia Jaekel

**Affiliations:** ^1^ Department of Psychology Ben‐Gurion University of the Negev Negev Israel; ^2^ Department of Psychology, Counselling and Therapy La Trobe University Melbourne Victoria Australia; ^3^ Clinical Sciences Murdoch Children's Research Hospital Melbourne Victoria Australia; ^4^ Newborn Research Royal Women's Hospital Melbourne Victoria Australia; ^5^ Department of Obstetrics, Gynaecology and Newborn Health University of Melbourne Melbourne Victoria Australia; ^6^ Department of Pediatrics University of Melbourne Melbourne Victoria Australia; ^7^ Department of Physiotherapy University of Melbourne Melbourne Victoria Australia; ^8^ The School of Brain Sciences and Cognition Ben Gurion University of the Negev Negev Israel; ^9^ Yale Child Study Center, School of Medicine Yale University New Haven Connecticut USA; ^10^ Department of Pediatrics University of California Irvine California USA; ^11^ Center for Newborn Research Children's Hospital of Orange County Orange California USA; ^12^ School of Psychology Sciences Monash University Melbourne Victoria Australia; ^13^ Unit of Psychology University of Oulu Oulu Finland; ^14^ Paediatrics I Essen University Hospital, University of Duisburg‐Essen Essen Germany; ^15^ Department of Psychology University of Warwick Coventry UK

**Keywords:** behaviour problems, cognition, fathers, individual participant data (IPD) meta‐analysis, preterm birth, sensitive parenting

## Abstract

**Background:**

Children born preterm (PT) face elevated neurodevelopmental risk, yet the role of fathers' sensitive caregiving in this process remains understudied.

**Objective:**

To test whether observed paternal sensitivity predicts cognitive and behavioural outcomes in PT children and moderates prematurity‐related risk.

**Methods:**

Individual participant data (IPD) from two prospective cohorts in Australia and Israel (2009–2014) were pooled (*N* = 207; 151 PT, 56 term). Father‐child and mother–child dyadic interactions at 6–12 months corrected age were observed and coded with the emotional availability (EA) sensitivity scale. Child cognition and behaviour were assessed at 5 years of age. Mixed‐effects regressions controlled for sex, age, multiple birth, small‐for‐gestational‐age status and maternal education.

**Results:**

Higher paternal sensitivity predicted higher cognitive scores (*β* = 0.25 per SD, 95% confidence interval = [0.09, 0.41], *p* < 0.003). Paternal sensitivity was not related to children's later behavioural problems (−0.003 [−0.21, 0.20], *p* = 0.981). No gestational age × paternal sensitivity interaction emerged.

**Conclusions:**

Sensitive fathering confers an additive, clinically meaningful benefit for cognitive function in both PT and term children, over and above social and medical risk. Early‐intervention programs that actively engage fathers and support sensitive responsive interactions may enhance developmental trajectories in this vulnerable population.

## Summary

- Sensitive father–child interactions in infancy are associated with better cognitive outcomes at preschool age.
- Paternal sensitivity appears to exert additive rather than buffering effects, suggesting that supportive caregiving benefits children across the full gestational range.
- Early parenting programs and NICU follow‐up services should move beyond mother‐only models and intentionally engage fathers as active caregivers.

## 1 Introduction

Children born preterm (PT: birth before 37 weeks of gestation) face increased risks of adverse developmental outcomes, including difficulties with cognitive functioning and higher prevalence of behavioural and emotional difficulties compared to their term‐born counterparts (Landau‐Crangle et al. 2018; Leoni et al. 2023; Linsell et al. 2016). These neurodevelopmental problems increase the risk for low academic achievement and mental health problems during adolescence and adulthood in PT‐born individuals (Clark and Woodward 2015; Lundequist et al. 2015). Understanding environmental factors that can foster optimal development among PT‐born children is critical for targeted early intervention and preventive care (Gueron‐Sela et al. 2015; Jaekel et al. 2015; Treyvaud et al. 2009; Vanes et al. 2021).

Sensitive parenting—characterized by parents' ability to accurately perceive, interpret and respond promptly and appropriately to children's cues (Ainsworth 1979; Joosen et al. 2013)—has been consistently associated with positive cognitive, social and emotional outcomes among typically developing children (Cooke et al. 2022; Foley et al. 2025). Maternal sensitivity, in particular, has been studied in relation to child developmental outcomes and has emerged as a protective factor that helps to mitigate risks associated with PT birth (Faure et al. 2017; Gueron‐Sela et al. 2015; Jaekel et al. 2015, 2026).

Although less studied, accumulating evidence underscores the unique and substantial contribution of fathers' sensitivity to child development (see Rodrigues et al. 2021 for a meta‐analysis). Fathers often engage differently with their children compared with mothers, frequently focusing on physical play, exploration and cognitive stimulation, potentially exerting unique influences on their child's developmental trajectory (Cabrera et al. 2014; Mills‐Koonce et al. 2015; Stgeorge and Freeman 2017). However, despite a steady increase in father involvement in childcare over the past three decades—evidenced by higher participation in parental leave and a growing share of caregiving responsibilities (Eydal and Rostgaard 2023)—the vast majority of parenting research continues to focus predominantly on mothers (Cabrera et al. 2018). The scarcity of studies examining the role of fathers is notable given recent evidence suggesting that fathers are becoming more engaged than in previous decades in the care of their PT infants, both in neonatal intensive care units (NICUs) and in the home environment (Stern‐Delfils et al. 2023). Thus, understanding paternal sensitivity and its impact on the developmental outcomes of children born PT is essential.

The limited research to date provides initial evidence of the important role of fathers' parenting behaviours in the context of PT birth. For example, Yogman et al. (1995) found that higher reported paternal involvement in childcare and play was associated with higher cognitive functioning in 3‐year‐old children born PT. To our knowledge, only one study has examined the longitudinal associations between observed paternal sensitivity and developmental outcomes in PT‐born children. This study demonstrated that higher observed paternal structuring and sensitivity were associated with improved cognitive and language development at 24 months, while greater paternal intrusiveness was linked to increased externalizing symptoms (McMahon et al. 2019).

The current study utilized IPD meta‐analysis methods to achieve three primary objectives: first, to evaluate the association between gestational age at birth and observed paternal sensitivity; second, to examine to what extent gestational age and paternal sensitivity predict cognitive, behavioural and emotional outcomes in children born PT; and third, to explore whether paternal sensitivity functions as a protective factor, moderating the developmental risks associated with PT birth. We proposed the following hypotheses:

1. Paternal sensitivity would be prospectively and positively associated with children's cognitive, behavioural and emotional outcomes across the whole range of gestation, while adjusting for known confounders (i.e., additive main effects), including infant biological sex, gestational age, birthweight, multiple births, SGA, maternal education and child age.
2. Gestational age will be negatively associated with children's outcomes.
3. An interaction between GA and paternal sensitivity is hypothesized, such that lower GA paired with low paternal sensitivity will correspond to the lowest cognitive functioning and highest behaviour problems. In contrast, high paternal sensitivity is anticipated to mitigate the adverse effects associated with lower GA.

## 2 Methods

The study was registered as an IPD meta‐analysis with the International Prospective Register of Systematic Reviews PROSPERO (Protocol # CRD42021256436).

### 2.1 Search Strategy and Study Selection Criteria

A two‐step process was applied to identify and select studies for inclusion. First, a systematic literature search was conducted in English in PubMed and Web of Science using the keywords ‘preterm’ OR ‘low birth weight’ AND ‘parenting behaviour/behaviour’ OR ‘observed parenting’ OR ‘parenting quality’ OR ‘parent–child dyad’ OR ‘parent–child interaction’ OR ‘parent–child relationship’ OR ‘synchrony’ OR ‘parental sensitivity’ AND ‘cognitive’ OR ‘neurodevelopment’ OR ‘IQ’ OR ‘social’ OR ‘socio‐emotional’ OR ‘behaviour/behaviour’ OR ‘internalizing’ OR ‘externalizing’ OR ‘mental health.’

**FIGURE 1:**
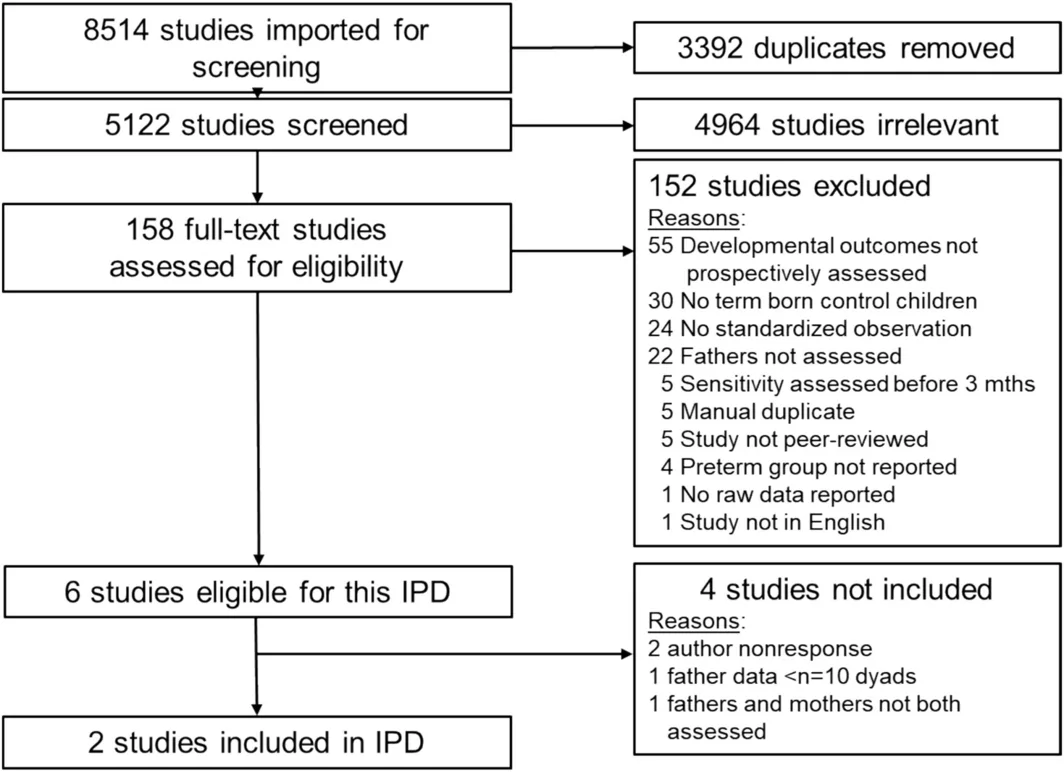
Flow diagram of study eligibility.

Inclusion criteria were as follows: (1) gestational age < 37 weeks and/or birth weight < 2500 g; (2) normal birth weight (> 2499 g) and/or term‐born (≥ 37 weeks) contemporaneous comparison group; (3) assessment of dyadic parent–child interactions with both mothers and fathers assessed separately at the same timepoint between age 3 months and 6 years using standardized behaviour observation and coding protocols; and (4) standardized assessment of child cognition and/or behaviour. Studies of selective cohorts (i.e., randomized controlled trials) were excluded. There was no restriction for year of birth, publication date or age at time of outcome assessment, but study design had to be prospective (i.e., assessment of sensitivity and later assessment of cognition and/or mental health). Only studies published in English were included. Title and abstract screening, duplicate removal, full‐text screening for eligibility and inclusion/exclusion decisions were carried out by two independent reviewers (JJ, KT) in Covidence. Conflicts were discussed and resolved jointly. Figure 1 shows a flow diagram of eligibility. By July 2022, six studies were identified; of these, two studies were deemed eligible for inclusion in the IPD harmonisation process based on similar constructs, definitions and measures used for the prospective assessment of both paternal and maternal sensitivity as well as child outcomes.

The authors of these publications were invited to participate in August 2022 by the seniour author (JJ). By April 2024, both cohort study teams had signed data‐sharing agreements with the University of Oulu, Finland.

Accordingly, the following two cohorts were included in the data harmonisation and pooling process:

- Israel: The Preterm Early Development Study (PEDS) included infants born in the Southern Region of Israel in 2009–2011 between 28 and 33 weeks gestation with low medical risk and birth weight > 1000 g (*n* = 86) and a term‐born comparison group (*n* = 37; Gueron‐Sela et al. 2015). At 6‐month corrected age (CA), both paternal and maternal sensitivity were assessed with the emotional availability (EA) scales (Biringen et al. 2014). Neurodevelopment was assessed at 12‐month CA with the Bayley Scales of Infant and Toddler Development‐Third Edition (Bayley 2006). At 5.5 years, children were tested with the cognitive processing screen scale from the Kaufman Assessment Battery for Children–Second Edition (K‐ABC‐II; Kaufmann and Kaufmann 2004), and parents rated behavioural and emotional symptoms with the Strengths and Difficulties Questionnaire (SDQ; Goodman 2001).
- Australia: The Victorian Infant Brain Studies 2 (VIBeS2) cohort included all livebirths < 30 weeks gestation at the Royal Women's Hospital in Melbourne in 2011–2014 (*n* = 65) and a term‐born normal birthweight comparison group (*n* = 19) (McMahon et al. 2019; Spittle et al. 2014). At 12‐month CA, both paternal and maternal sensitivity was assessed with the EA scales (Biringen et al. 2014). Neurodevelopment was assessed at 24‐month CA with the Bayley‐III (Bayley 2006). At 5 years, children were administered the Wechsler Preschool and Primary Scale of Intelligence–Fourth edition (WPPSI‐IV; Wechsler 2012) full‐scale IQ test, and parents rated behavioural and emotional symptoms with the SDQ (Goodman 2001).

### 2.2 Data Extraction, Harmonisation and Analysis

Participating groups (*n* = 2) had obtained ethical permissions for the original data collection themselves. They completed a data‐sharing agreement with the University of Oulu, Finland, to transfer nonidentifiable individual‐level data for the analysis. Groups were requested to provide infant neonatal and demographic data (e.g., infant gestational age at birth [weeks], birth weight [grams], sex [binary coded], parents' education), scores for paternal sensitivity and standardized neurodevelopmental, cognitive and behavioural outcome data.

Data was harmonized in SPSS 27. All observation and test scores were *z‐*standardized according to each cohort‐specific contemporaneous term‐born comparison group before pooling. Participants with standardized neurodevelopmental test scores deviating more than 2 SD below their respective comparison group's mean were binary coded as having a neurodevelopmental delay or impairment. Because information about paternal education was not complete, data on maternal education was used as a proxy and binary coded according to the International Standard Classification of Education (ISCED; UNESCO 2012) into medium/low versus high = ‘completion of upper secondary education, high school degree or equivalent, allowing unrestricted university entrance, ≥ ISCED 3.’ Small for gestational age (SGA, < 10. centile by sex) birth was coded according to Fenton (Fenton and Kim 2013).

One‐stage IPD meta‐analyses were performed as mixed‐effects linear regressions in Stata 17 (StataCorp LLC; Texas, USA). Models were controlled for fixed effects of infant sex, age at outcome assessment, multiple birth, SGA birth and maternal education, as well as the nestedness of data in cohorts (i.e., including a random effect representing study site). To test H1, the fixed and random effects of gestational age on paternal sensitivity in dyadic interactions are reported. Next, we report the fixed main effects of gestational age and paternal sensitivity on developmental outcomes, along with confounders (H2). Finally, we tested the fixed interaction effects of gestational age by paternal sensitivity on cognition and behaviour (H3). As sensitivity analyses, we tested the associations of gestational age at birth and dyadic paternal sensitivity with cognition and behaviour both with and without additionally controlling for maternal sensitivity and the presence of child neurodevelopmental impairment.

## 3 Results

Individual cohort sample sizes ranged from 84 to 123 families (Table 1). There was substantial variation between cohorts on all variables. Both studies had assessed maternal sensitivity in parallel to paternal sensitivity during dyadic interactions, and maternal and paternal behaviours were significantly correlated *r* = 0.19 (*p* < 0.001).

**TABLE 1:** Descriptive characteristics by cohorts included in IPD sample (preterm/term‐born samples).

	PEDS, Israel (*n* = 86/37)	VIBeS2, Australia (*n =* 65/19)
**Year(s) of birth**	2009–2011	2011–2014
**Female sex**	46.3%/53.7%	52.5%/53.6%
**Gestation (weeks)**	33.1/39.4	27.4/39.3
**Birthweight (g)**	1913/3329	1033/3395
**Multiple birth**	0.0%/0.0%	40.6%/1.0%
**SGA birth** ^a^	25.6%/13.4%	11.3%/0.0%
**Mothers with upper secondary education** ^b^	77.7%/88.1%	96.7%/100.0%
**Mother's age (years)**	31.0/30.5	32.8/32.7
**Neurodev. <** − **2SD** ^c^	6.2%/4.8%	1.4%/8.0%
**Paternal sensitivity** ^c^	−0.18/−0.10	−0.04/0.17
**Maternal sensitivity** ^c^	−0.28/0.19	−0.08/0.10
**Cognition** ^c^	−0.14/0.05	−0.30/−0.14
**SDQ total score** ^c^	0.73/0.00	0.53/−0.18

*Note:* values are reported as means or % depending on variable scaling.

Abbreviations: PEDS=Preterm Early Development Study; SDQ = Strengths and Difficulties Questionnaire; SGA = small for gestational age at birth; VIBeS2 = Victorian Infant Brain Studies 2.

^a^ Coded based on Fenton chart for original gestation, birthweight and sex study data.

^b^ Harmonized according to the International Standard Classification of Education (ISCED ≥ 3, completion of upper secondary education with unrestricted university entrance qualification).

^c^ Values *z*‐standardized according to cohort‐specific healthy term‐born controls in the total sample, before removing participants with missing data.

### 3.1 One‐Stage IPD Meta‐Analysis

The fixed effect of gestation at birth on paternal sensitivity, adjusted for child age, sex, multiple and SGA birth, and mother's education, was not significant (*coefficient* = 0.001 per week, 95% confidence interval (CI) = [−0.03, 0.03], *p* = 0.930). The random effect of gestation was estimated at *SD* = 0.00 [0.00, 0.00], indicating little variation between cohorts in the relationship between these two variables.

**FIGURE 2:**
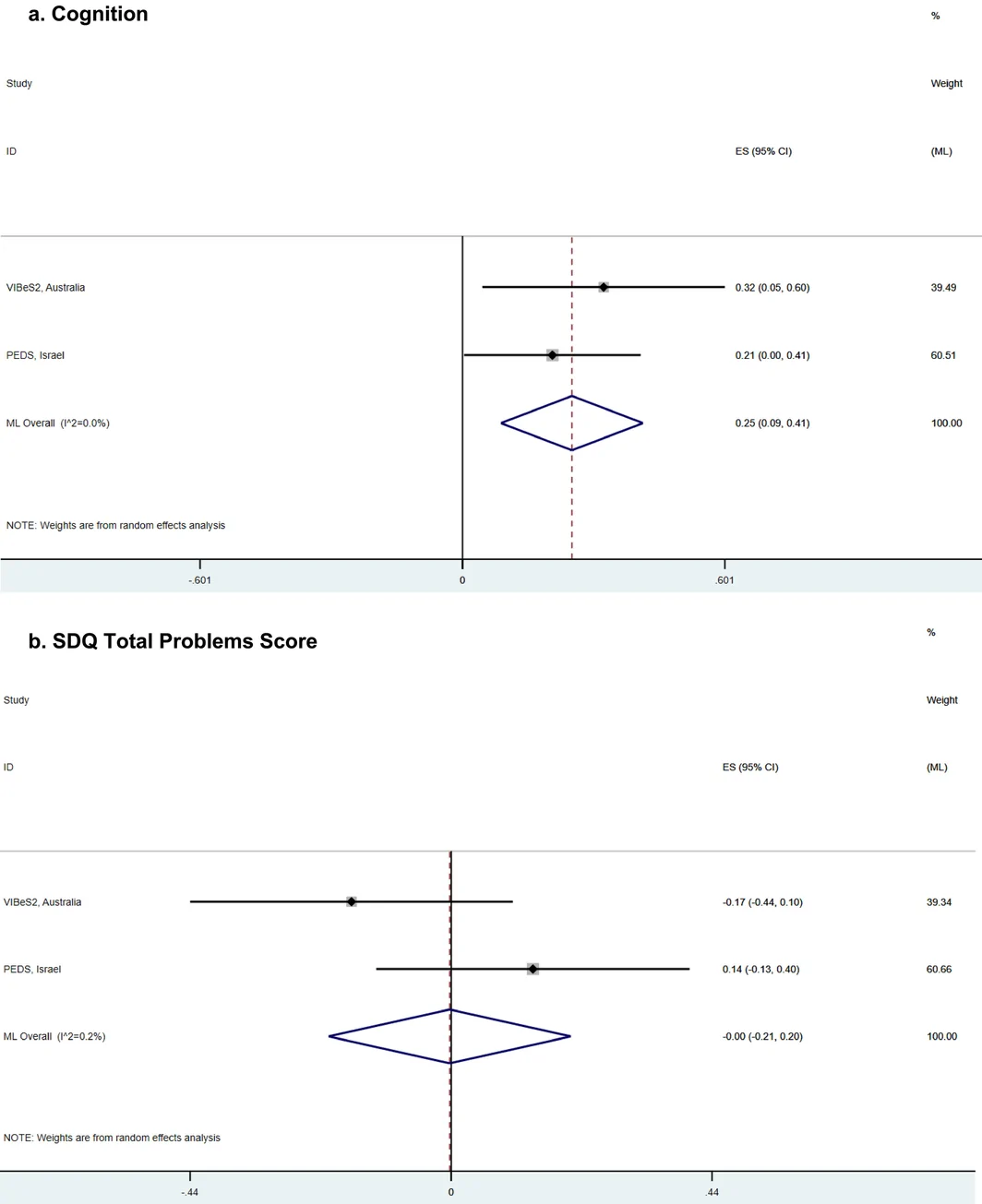
Forest plots showing adjusted one‐stage IPD meta‐analysis effects of paternal sensitivity (coefficients and 95% CIs) on child cognition and behaviour. *Note:* Weights reflect individual study *n* while accounting for within‐study variability and between‐study variance, adjusted for fixed effects of infant sex, age at outcome assessment, multiple birth, SGA birth, maternal education and random effects of gestational age and paternal sensitivity.

Table 2 shows the fixed effects of gestation at birth (cognition: *coefficient* = 0.02 per week, 95% CI = [−0.02, 0.06], *p* = 0.336; behaviour problems: −0.07, 95% CI [−0.12, −0.02], *p* < 0.003) and paternal sensitivity (cognition: 0.25 per SD, 95% CI [0.09, 0.41], *p* < 0.003; behaviour problems: −0003, 95% CI [−0.21, 0.20], *p* = 0.981) on child cognition and behaviour problems across cohorts, adjusted for child sex, age at outcome assessment, multiple birth, SGA birth and maternal education. Figure 2 displays the forest plot of the effect of dyadic sensitivity on child cognition and behaviour by cohorts.

**TABLE 2:** One‐stage IPD mixed‐effects model results showing associations of paternal sensitivity, child gestational age, and other covariates with cognition and behaviour.

Dependent variable	Cognition (dyad *n =* 195; cohort *n =* 2; dyad avg. per cohort = 98) coefficient (95% CI)	SDQ total problem score (dyad *n* = 183; cohort *n* = 2; dyad avg. per cohort = 92) coefficient (95% CI)
*Fixed effects*		
Paternal sensitivity (per SD)	0.25 (0.09, 0.41)**	−0.003 (−0.21, 0.20)
Gestation (per week)	0.02 (−0.02, 0.06)	−0.07 (−0.12, −0.02)**
Female sex	0.15 (−0.61, 0.50)	−0.37 (−0.72, −0.01)*
Child age (per month)	0.08 (−0.003, 0.16)	0.01 (−0.08, 0.11)
High mother's education	−0.05 (−0.61, 0.50)	−0.18 (−0.84, 0.47)
Multiple birth	0.53 (0.02, 1.03)*	−0.42 (−1.01, 0.18)
SGA birth	0.13 (−0.26, 0.53)	0.47 (0.01, 0.93)*
*Random effects by cohort*		
SD (gestation)	0.00 (0.00, 0.00)	0.00 (0.00, 120501.00)
SD (paternal sensitivity)	0.00 (0.00, 0.00)	0.05 (0.00, 333.56)
SD (residual)	1.06 (0.96, 1.17)	1.21 (1.09, 1.35)
*Log‐likelihood*	−287.98	−295.49

* *p* < 0.05.

** *p* < 0.01.

The fixed interaction effects of gestation by sensitivity on cognition and behaviour were not significant (i.e., cognition −0.01, 95% CI [−0.04, 0.02], *p* = 0.512; behaviour problems: −0.01, 95% CI [−0.03, 0.05], *p* = 0.537).

We ran three sets of sensitivity analyses. First, we tested the associations of gestation at birth and dyadic paternal sensitivity with cognition and behaviour while additionally controlling for maternal sensitivity. When competing with each other, none of the effects of paternal nor maternal sensitivity in dyadic interactions significantly contributed to later child cognition and behaviour (e.g., paternal sensitivity on cognition: 0.39, 95% CI [−0.70, 1.49], *p* = 0.482; maternal sensitivity on cognition: 0.14, 95% [−0.01, 0.29], *p* = 0.063), after controlling for confounders.

Second, we tested the associations of gestation at birth and dyadic paternal sensitivity with cognition and behaviour, while additionally controlling for child neurodevelopmental impairment and confounders. Effects remained stable and similar in size (e.g., paternal sensitivity on cognition: 0.25, 95% CI [0.09, 0.41], *p* = 0.002; paternal sensitivity on behaviour problems: −0.01, 95% CI [−0.21, 0.19], *p* = 0.930).

Finally, effects of gestation and paternal sensitivity on cognition and behaviour remained similar when confounding variables (i.e., child sex, age at outcome assessment, multiple birth, SGA birth and maternal education) were not included (e.g., paternal sensitivity on cognition: 0.26, 95% CI [0.10, 0.42], *p* = 0.001; paternal sensitivity on behaviour problems: −0.06, 95% CI [−0.30, 0.19], *p* = 0.658).

## 4 Discussion

This individual participant data (IPD) meta‐analysis is the first to examine the role of paternal sensitivity in shaping cognitive and behavioural outcomes among children born PT across two international cohorts. Our findings provide evidence that higher paternal sensitivity during early father‐child interactions is associated with higher cognitive functioning in PT‐born children. Importantly, this effect remained robust after adjusting for key covariates, including maternal education, neurodevelopmental impairment and multiple birth status. In contrast, we did not find a significant association between paternal sensitivity and children's behavioural and emotional difficulties, nor evidence for an interaction between paternal sensitivity and gestational age in predicting child outcomes.

The positive association between paternal sensitivity and children's cognitive functioning aligns with previous findings in neurotypical and at‐risk populations, highlighting sensitive fathering as a potentially critical contributor to early cognitive growth (McMahon et al. 2019; Rodrigues et al. 2021). Our results extend this evidence base by demonstrating that this association holds in the context of PT birth. These findings suggest that fathers may provide cognitive stimulation or scaffolding that may be beneficial for most children, including those children born PT—who often face specific vulnerabilities in motor skills, executive functioning and problem solving, all of which serve as critical foundations for broader cognitive growth (Brumbaugh et al. 2014; Sandoval et al. 2022). Father‐child interactions are often characterized by high‐energy physical play, managed risk, cognitive novelty and problem‐focused dialogue, which can work together to strengthen children's gross‐motor coordination and the cognitive systems that underlie exploration, problem‐solving and eventually academic success (Paquette 2004). Indeed, sensitive parenting has been associated with better executive functioning in children (Valcan et al. 2018), and further research investigating the specific relationships between paternal parenting and cognitive development for children born PT may provide information that can be used for targeted interventions to promote optimal outcomes.

When maternal and paternal sensitivity were included in the same model, neither showed a unique association with children's cognitive outcomes. This is likely because parents within the same family tend to exhibit similar caregiving behaviours due to factors such as assortative partnering, shared environments and exposure to similar stressors (Deneault et al. 2022). Accordingly, the correlation between maternal and paternal sensitivity was statistically significant, albeit modest. This also aligns with prior work showing that parents share some variance in caregiving but also individually differ in their interactions with the child (Deneault et al. 2022). These patterns likely reflect both shared contextual influences (e.g., co‐parenting climate) and distinct parental roles and interaction styles. Consequently, maternal and paternal sensitivity share substantial variance, and once this overlap is accounted for, the unique predictive contribution of each parent may become nonsignificant, also due to statistical multicollinearity that makes models less stable. Importantly, this pattern should not be interpreted as indicating that fathers do not matter; rather, it suggests that mothers and fathers jointly contribute to a shared sensitive caregiving climate that supports children's cognitive development, making it difficult to statistically disentangle their individual effects within the parenting triad.

In contrast, paternal sensitivity was not associated with children's behavioural and emotional difficulties. This finding diverges somewhat from the broader parenting literature, in which both mothers' and fathers' sensitivity have been linked to lower levels of externalizing and internalizing symptoms (Cooke et al. 2022; Mesman et al. 2009; Jokiranta‐Olkoniemi et al. 2026). One possible explanation is that paternal sensitivity and child behaviour problems may reciprocally consolidate one another over time, which could lead to stronger associations as children age (Rodrigues et al. 2021). Additionally, other aspects of fathering, such as discipline style (McKee et al. 2007), involvement in daily caregiving (Zhang et al. 2021), paternal playfulness (Menashe‐Grinberg and Atzaba‐Poria 2017) and co‐parenting dynamics (Gueron‐Sela et al. 2015) may be more relevant for socio‐emotional functioning than sensitivity alone.

In the current study, gestational age was not directly associated with paternal sensitivity. Prior research has reported mixed findings regarding this association: some home‐ and lab‐based studies document lower sensitivity or reduced father–infant synchrony among fathers of preterm infants (Feldman and Eidelman 2007; Harrison and Magill‐Evans 1996), whereas larger observational studies have found negligible prematurity effects or patterns that vary across contexts (Hall et al. 2015). Taken together, these inconsistencies suggest that the link between gestational age and paternal sensitivity is likely more complex than a simple linear association and may depend on factors such as infant medical risk, the developmental timing of assessments and fathers' interpretations or perceptions of their infant's vulnerability. In addition, differences from prior IPD findings showing links between maternal sensitivity and gestational age (Jaekel et al. 2025) may reflect distinct caregiving roles and involvement patterns in early infancy. It is also important to note that the present sample comprised relatively low‐risk preterm infants, which may have attenuated variability in caregiving responses to prematurity. Finally, given the modest sample size and inclusion of only two cohorts, the current study may have been underpowered to detect small associations between gestational age and paternal sensitivity.

Additionally, contrary to our hypothesis, paternal sensitivity did not interact with gestational age in predicting child outcomes, suggesting that its beneficial effects may operate in an additive rather than a conditional manner with respect to the degree of prematurity. This finding contrasts with prior theoretical frameworks (Hartman and Belsky 2018) and empirical evidence (Faure et al. 2017; Gueron‐Sela et al. 2015; Jaekel et al. 2015, 2026) positing parental sensitivity as a protective factor that buffers PT infants against neurodevelopmental risk. However, it is consistent with other studies reporting no moderating effects of parental characteristics or behaviours on developmental outcomes among children born PT (Gould et al. 2022; Oosterom et al. 2020; Jokiranta‐Olkoniemi et al. 2026). A recent scoping review on PT birth and low birth weight as environmental sensitivity factors further highlighted mixed evidence across studies, potentially reflecting methodological differences in sample size, timing of assessments, measurement approaches and statistical modelling (Lionetti et al. 2025). Moreover, findings from an IPD meta‐analysis of over 30 000 children demonstrated that interactions between child characteristics (including birthweight) and parenting factors seldom replicate across cohorts or developmental domains. Instead, the most robust evidence supports additive effects, indicating that child characteristics and parenting behaviours are consistently, but largely independently, associated with developmental outcomes (Eves et al. 2025). The current study aligns with this growing body of evidence, underscoring that while parenting behaviours are crucial for supporting the development of PT infants, their effects may not depend on the degree of prematurity.

While the current study has several strengths, including the use of a harmonized IPD approach that increases statistical power and generalizability and the inclusion of observational assessments of parenting across two independent cohorts, some limitations should be acknowledged. First, although IPD methods increase statistical power and precision compared to traditional meta‐analyses, the number of included studies was small (two cohorts), and sample sizes remained modest. This may have limited our ability to detect small or interaction effects, particularly those hypothesized between gestational age and paternal sensitivity. In addition, the relatively small size of the term‐born comparison group in the Australian cohort may have limited the precision of cohort‐specific standardization, potentially reducing variability in the reference distribution and introducing noise into the harmonized scores. Finally, heterogeneity across cohorts in measurement timing and outcome could have affected the pattern of results. While careful standardization procedures were applied, and similar measures were used in both cohorts to assess paternal sensitivity and child behaviour problems, cohort‐specific contexts may also have introduced noise or attenuated effect estimates. For instance, there was no association between gestational age and cognitive skills, which may be explained by a nonsignificant negative correlation between gestation and cognitive skills in the Australian term‐born children, of whom 8% had neurodevelopmental impairments in infancy. This possible recruitment bias may have introduced statistical artefacts that limit the generalizability of the current findings. These biases also include that there was almost no variation in maternal education in the Australian sample, and little variation in Israel too.

In conclusion, this IPD meta‐analysis provides novel and robust evidence that sensitive fathering is a promotive factor for cognitive development among children across a wide range of GA. These findings have several important implications for clinical practice and early intervention. First, they underscore the value of including fathers in early parenting interventions, particularly those targeting cognitive stimulation and parent–child interaction quality. Historically, most interventions have focused on mothers, especially in the context of NICU follow‐up or developmental risk (Lavallée et al. 2021). Our results highlight the need to engage fathers not only as passive observers but as active agents in their children's cognitive development. Second, given the observed independent contribution of paternal sensitivity, parenting programs should consider tailored components that support fathers' unique interaction styles, such as play‐based engagement or scaffolding cognitive skills. At the same time, such programs may also benefit from encouraging both mothers and fathers to incorporate a broader range of interaction styles, including these forms of engagement in combination with warm, nourishing stimulation. This may support children's cognitive development in ways that encompass the whole continuum of gendered interaction styles.

## Author Contributions

**Noa Gueron‐Sela:** writing – original draft, data curation, resources. **Karli Treyvaud:** conceptualization, data curation, writing – review and editing, methodology, resources. **Naama Atzaba‐Poria:** writing – review and editing, data curation, resources. **Jeanie Cheong:** data curation, resources, writing – review and editing. **Alicia Spittle:** writing – review and editing, data curation, resources. **Tal Yatziv:** data curation, resources, writing – review and editing. **Peter J. Anderson:** conceptualization, writing – review and editing, data curation, resources. **Julia Jaekel:** conceptualization, methodology, formal analysis, project administration, writing – original draft, visualization, data curation, resources, investigation.

## Funding

While each of the study cohorts included here received their own funding for data collection and analyses, this IPD research did not receive any specific grant from funding agencies in the public, commercial or not‐for‐profit sectors.

## Ethics Statement

Participating groups had obtained ethical permissions for the original data collection themselves (VIBeS: Royal Children's Hospital Human Research Ethics Committee #26088; PEDS: Helsinki Review Board at Soroka University Medical Center #1033). They completed a data sharing agreement with the University of Oulu, Finland, to transfer nonidentifiable individual level data for the analysis.

## Consent

All participants were required to provide written informed consent.

## Data Availability

Due to ethical restrictions, the raw data files will not be shared.
